# Landscape Is the Main Driver of Weed Assemblages in Field Margins but Is Outperformed by Crop Competition in Field Cores

**DOI:** 10.3390/plants10102131

**Published:** 2021-10-07

**Authors:** Adrien Berquer, Olivier Martin, Sabrina Gaba

**Affiliations:** 1 Centre d’Etudes Biologiques de Chizé, UMR7372, CNRS & La Rochelle Université, 79360 Villiers-en-Bois, France; adrien.berquer@cebc.cnrs.fr; 2 UR 0546 Biostatistiques et Processus Spatiaux, INRAE, CEDEX 9, 84914 Avignon, France; olivier.martin@inrae.fr; 3 LTSER, «Zone Atelier Plaine & Val de Sèvre», 79360 Villiers-en-Bois, France; 4 Centre d’Etudes Biologiques de Chizé, USC 1339 Agripop, INRAE, 79360 Villiers-en-Bois, France

**Keywords:** agroecology, competition, dispersal, landscape, oilseed rape, sustainable weed management

## Abstract

Weeds are considered a major pest for crops, and as such have been intensively managed by farmers. However, weeds, by providing resources, also support farmland biodiversity. The challenge for sustainable weed management is therefore to maintain weed diversity without compromising crop production. Meeting this challenge requires determining the processes that shape weed assemblages, and how agricultural practices and landscape arrangement affect them. In this study, we assess the effects of crop competition on weeds, nitrogen input, weed control and landscape on both weed diversity and abundance in the margins and centres of 115 oilseed rape fields in Western France. We show that weed assemblages in field cores were mainly shaped by crop height, a proxy of crop competition. By contrast, weed assemblages in field margins increased with the number of meadows in the landscape, revealing the role of spatial dispersal. Using structural equation modelling, we further show that in the field core, weed assemblages were also indirectly shaped by landscape through spatial dispersal from the field margin. Overall, our study gives empirical support for crop competition as a way to reduce the intensity of chemical weeding, and for meadows as a way to enhance biodiversity in the landscape.

## 1. Introduction

Taking into account the challenges of sustainable food for a growing human population, the preservation of biodiversity and natural resources and the mitigation of climate change requires a profound transition in our agricultural and food system [1,2]. Weed management in arable crops is typical of this issue. Weeds are recognized as a major pest in agriculture, resulting in yield loss of up to 30% [3]. For decades, they have been intensively managed to reduce their competition for resources with crop plants. This has resulted in the decline of at least 20% of weed species over the past 30 years [4], and an overall decline in rare flagship species [5]. However, by providing food and shelter for birds, insects and small mammals [6,7], weeds are also an important component in the maintenance of farmland biodiversity and agroecosystem functioning [8,9]. To meet agricultural production demand while conserving weed diversity and enhancing its related ecological functions, promotion of diverse weed assemblages has been suggested, assuming that increasing species richness would ensure for weed functions without selecting for few dominant species [10,11]. Designing management strategies that ensure for diverse weed assemblages therefore requires strengthening our understanding of the processes that shape weed species richness and abundance.

Weed species assemblages can be understood in terms of a complex scheme including interactions between ecological processes (e.g., competition, spatial dispersal) operating over various scales and management through disturbance regimes (e.g., weeding operations) and resource levels (e.g., light, nitrogen) [12,13,14,15,16]. While there is substantial evidence showing that crop type and farming practices influence weed species richness [17,18,19], weed abundance [20,21], or crop–weed competition [22], only recently have studies explored the interactive effects of competition and farming practices on weed assemblages [23]. Crop competition has however been acknowledged as a way to regulate weed species [24]. The effect of landscape on weed abundance is also less documented compared to weed species richness, and when studied was shown to have either no effect [25] or an indirect effect [26] through an interaction with farmer management intensity. Indeed, evidence on the interplay of local and landscape effects on weeds have recently been revealed [13,25,27]. For instance, Henckel et al. [28] demonstrated that the presence of organic farming in the surrounding landscape of conventional fields could balance the negative effect of conventional management through species dispersal. The diversity of crop types [12] and the amount of seminatural habitats [29,30] in the landscape also benefit in-field weed species richness. However, the effect of landscape varies with field position (i.e., field core versus field margin [12,13,31]) revealing the complex interplay between spatial dispersion and local processes. These differences can indeed be attributed to the variation in farming practices (crop density, fertilization and weed control) as well as to their distances to source habitats. However, whether landscape effects interact with competition with crop plants, disturbances induced by weed control or both remains to be established.

In this study, we evaluated the interactive effects of crop–weed competition, farming practices and landscape on both weed diversity and abundance in the margins and centres of 115 oilseed rape (*Brassica napus* L.) fields in South-West France. We used a new approach to evaluate the effects of landscape variables without specifying *a priori* distances of spatial extents of their effects [32]. To our knowledge, this is the first study to address the combined effects of competition, farming practices and landscape on both weed species richness and abundance in the margins and centres of arable fields, considering that the spatial extent of the landscape variables can vary with the landscape variables, the weed metrics and the field compartment. As a first step, we assessed the effects of competition, farming practices and landscape on weed species richness and abundance in the two field compartments. Then, we investigated whether local dispersal from field margin to field core could compensate for a loss of weed diversity through an indirect effect of spatial dispersal from the landscape, as highlighted by Bourgeois et al. [13]. We expected the contribution of competition to be higher in field cores due to a higher crop density. We also expected the contribution of competition to increase with the amount of nitrogen, because oilseed rape plants are nitrophilous plants [33], and decrease with higher weed management due to the selection of specialist species [34]. We further expected a higher response of weed abundance to competition compared to weed species richness, especially in field cores. Finally, we expect landscape effect to act predominantly indirectly across the field margin on in-field weed assemblages.

## 2. Results

A total of 158 weed species was identified across the 115 oilseed rape fields sampled from 2014 and 2018. We identified 131 weed species in the field cores and 143 in the field margins. *Mercurialis annua* L. was the most abundant and common weed species occurring in 92 fields. A total of 90 species (57.0% of all species) occurred in fewer than 10% of the sampled fields. Mean species richness per field was 28.85 ± 8.73 (min = 10, max = 53) species, and mean abundance was 267.60 ± 150.97 (min = 48, max = 956). Weed species richness was on average higher in the field margin, with 19.97 ± 8.11 (min = 3, max = 42) species, than in field core with an average of 11.09 ± 4.89 (min = 3, max = 40) species when accounting for the same sampling effort using a 5000 times bootstrap of five quadrats in the field core. In the same way, the abundance was higher in the field margin (88.84 ± 45.47) than in field core (48.72 ± 33.18; on average in five quadrats using a bootstrap). Crop height was significantly lower in the field margin (54.4 cm ± 47.1 cm) than in the field core (145.9 cm ± 23.7 cm; Wilcoxon paired-test, V = 17, *p*-value < 0.001). In 3.5% of the fields, there was no crop plant in the margin.

### 2.1. Competition and Weed Management Highly Affect Weed Species Richness in Field Core

**Figure 1:**
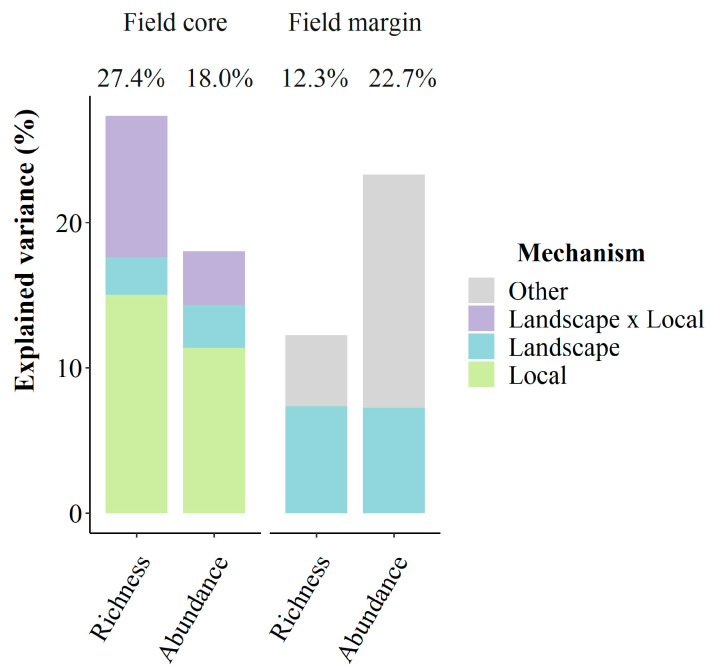
Percentage of explained variance by local factors (crop height, the amount of nitrogen, the intensity of herbicide use and number of mechanical operations), landscape (amount of organic farming, meadows, oilseed rape and hedge density) and weather conditions (rainfall and temperature) on weed species richness and abundance in field cores and field margins. The intensity of herbicide use is expressed using the herbicide TFI. The buffer radii at which the amount of each landscape variable was estimated are shown in Table 1 for field cores and Table 2 for field margins. R-squared computed from the type III ANOVAs of respective models are indicated above each corresponding bar plot.

**Figure 2:**
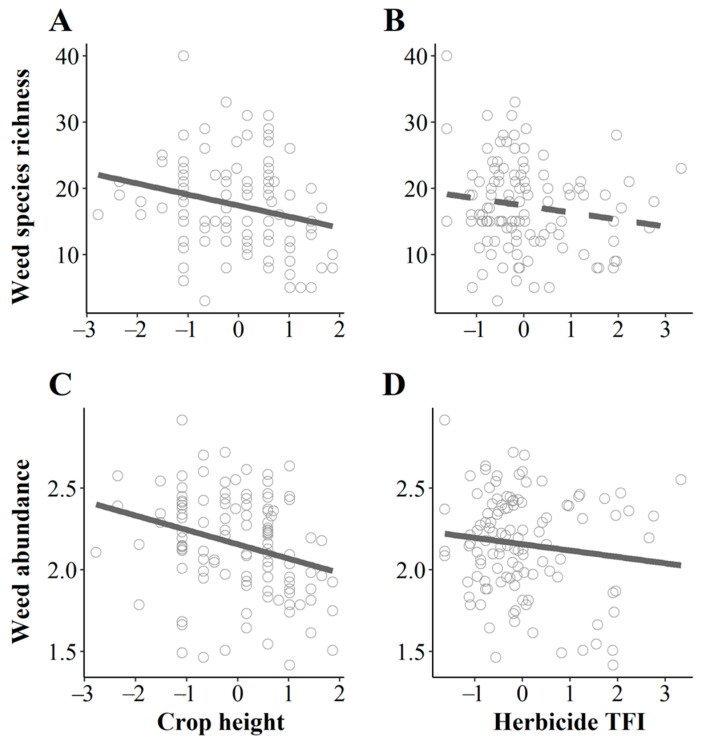
Relationship between weed species richness (**A**,**B**) and abundance (**C**,**D**) in field cores with crop height (**A**,**C**) and the intensity of herbicide applications (**B**,**D**). Abundance was log-transformed and explanatory variables were scaled. The intensity of herbicide use is expressed using the Treatment Frequency Index. Dashed line indicates a nonsignificant relationship.

For weed species richness, the selection procedure retained the variables related to competition (crop height and nitrogen) and chemical disturbances as well as the interaction between nitrogen and herbicides (Table 1A). These effects explained 15% of the variance of weed species richness in field cores when using Treatment Frequency Index (TFI) as a proxy of herbicide use (Figure 1; 11% with amount of herbicide active substances (QA) Figure S1). Weed species richness significantly decreased with crop height (Figure 2A) but not with herbicides (Figure 2B) nor with nitrogen. Contrary to our expectation, we did not find any significant interaction between crop height and the amount of nitrogen or the quantity of herbicide use. Rather, we found a significant positive effect of the ‘nitrogen × herbicides’ interaction on weed species richness, suggesting a higher efficiency of weed chemical control in nitrogen-rich fields.

Adding landscape variables improved the model, which explained 27.4% of the variance (Figure 1; 22.4% with QA Figure S1). However, the contribution of landscape variables alone to weed species richness was lower compared to the contribution of the local variables. The estimated spatial extent of the effects of the landscape variables was always lower than 1000 m, ranging from a very small scale for hedge density (17 m from the border of the field), to medium scales for meadows (500 m), oilseed rape (600 m) and organic farming (780 m). Weed species richness was generally unaffected by landscape variables. The interplay between local and landscape variables was revealed by the significant interaction between the number of meadows and amount of nitrogen (Table 1 and Table S1), suggesting a lower positive effect of nitrogen on weed species richness in fields surrounded by a high number of meadows. We also found a significant positive interaction between the amount of organic farmed fields and herbicides when using TFI as a proxy of herbicide intensity (Table 1; i.e., the relationship is almost significant with QA, *p* = 0.054; Table S1). This suggests that herbicide use significantly decreased weed species richness in oilseed rape fields in landscapes rich in organic farming.

### 2.2. Competition and Weed Management Strongly Affect Weed Abundance in Field Cores

The pattern for weed abundance in field cores was mostly consistent with the pattern of weed species richness: environmental variables and mechanical weed control were discarded, as was nitrogen (Table 1B). Weed abundance in the centre of oilseed rape fields significantly decreased with crop height (Figure 2C) and herbicide use (Figure 2D), with a higher effect attributed to crop height. Adding the landscape variables improved the model (Figure 1), although we found no significant effect of landscape variables on weed abundance (Table 1B).

### 2.3. Landscape Is a Major Driver of Weed Assemblages in Field Margins

**Figure 3:**
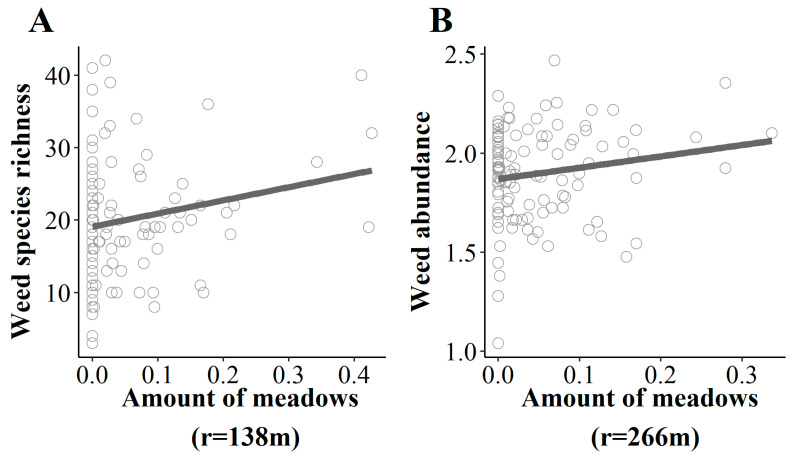
Relationship between weed species richness (**A**) and abundance (**B**) in field margins with the number of meadows in the surrounding landscape at respective buffer radii of 138 and 266 m. Abundance was log-transformed.

Diversity and abundance patterns showed a contrasted situation in field margins, revealing that weed species assemblages in field margins were mainly affected by environmental conditions and landscape (Figure 1). The selection procedure removed crop height and farming practices for both weed species richness and abundance, while several environmental variables were kept. Weed species richness and abundance significantly decreased with rainfall (Table 2A), while only weed abundance increased with temperature (Table 2B). Landscape effect was mainly due to the number of meadows, which had a significant positive effect on both weed species richness (Figure 3A) and weed abundance (Figure 3B) at small scale, i.e., for 140 m from the border of the field for weed species richness and 265 m for weed abundance.

### 2.4. Multiscale Processes Shape Weed Assemblages in Field Cores

**Figure 4:**
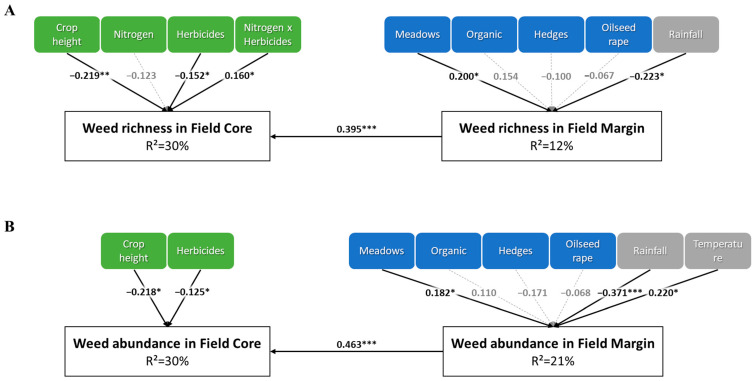
Structural Equation Models for weed species richness (**A**) and abundance (**B**) where the link between field margin and field core was specified. Arrows represent the directionality of the effect, and the coefficients indicate the standardized estimates. Dashed lines and grey estimates represent nonsignificant effects. *: *p*-value < 0.05; **: *p*-value < 0.01; ***: *p*-value < 0.001. FC: field core, FM: field margin. The intensity of herbicide use is expressed using the Treatment Frequency Index. The buffer radius at which the amount of each landscape variable was estimated is shown in Table 2 for field margin.

Because landscape affects weed assemblages in field margins and previous studies revealed local dispersal from field margins to field cores, we performed an SEM to assess the joint effect of local and landscape processes when considering weed assemblages in the two field compartments. The best model shown by BIC-based selection for both weed species richness and abundance was the SEM considering an indirect effect of landscape on weed assemblages in the field core through to the field margin. Competing models with either a direct link between landscape variables and weeds in field cores, or no link between the margin and centre of the fields were never retained (Tables S2 and S3). Accounting for local dispersal from the field margin strongly increased the part of variance explained in the field core, with R-squared increasing from 27% to 30% for weed species richness and from 18% to 30% for weed abundance using TFI (22.4% to 33% and 16% to 32% when using QA). The strength of local dispersal was similar for weed species richness and abundance (Figure 4). These analyses suggest that weed assemblages in the centre of oilseed rape fields were shaped by local factors (mainly crop competition and chemical weeding) and local dispersal from field margins, its relative importance being related to the number of meadows in the surrounding landscape. Interestingly, when accounting for spatial dispersal across the field margin, herbicide applications had a significant negative effect on both species richness and abundance. This effect was found when using linear models (without incorporating spatial dispersal across the field margin) for analysing weed abundance in the field core, but this was not the case for weed species richness. These results are, however, in line with the significant positive interaction between herbicide use and amount of organically farmed fields and suggest that herbicides decrease weed species richness in oilseed rape fields located in more diversified landscapes (i.e., a higher number of meadows and amount of organic farming).

## 3. Discussion

Weed species assemblages are the result of the complex interplay between weed–crop competition, farming practices and landscape. In this study, we aimed at determining their relative contribution on both weed species richness and abundance in the margins and centres of 115 oilseed rape fields. As expected, our results highlighted that the mechanisms shaping weed assemblages differed between field cores and margins. Using crop height as a proxy for weed–crop competition, we found that competition strongly affected weed species richness and abundance in field cores while low or no effects could be detected for farming practices and landscape. Conversely, crop competition had almost no effect on weed assemblages in field margins, where we found a strong effect of landscape, suggesting a predominant role of spatial dispersal. Although landscape had no direct effect on weed species richness and abundance in field cores, the use of structural equation modelling revealed that landscape arrangement may affect weed assemblages in field cores indirectly through field margins.

As expected, the main driver of weed assemblages in field cores was the presence of the crop itself, since its height was positively related to a decrease of weed species richness and abundance. Competition with the crop had a higher effect on weed abundance than on weed species richness. Taller and denser crop plants in field cores are more prone to take up resources as light and nutrients, leaving lower amounts of resources available for weeds [35]. These results confirm the competitive ability of oilseed rape against weeds [36,37,38], but to our knowledge, our study is the first to demonstrate it in oilseed rape farmers’ fields and with a natural flora. We expected a higher importance of competition in high nitrogen conditions because oilseed rape plants are nitrophilous plants and because reduced nitrogen amount might delay canopy closure [39]. No effect of nitrogen alone or in interaction with crop height was found, however. While mechanical weeding did not affect weed assemblages in field cores, we found a significant decrease of weed abundance with herbicide use, although the effect was lower compared with the effect of the competition with crop plants. We also found a significant negative effect of herbicide applications on weed species richness, but only when interacting with landscape or accounting for spatial dispersal across the field margin. Herbicide application directly affects weeds and is generally related to a decrease in weed species richness, mostly due to removal of rare species [40,41]. Fried et al. [34] found that weed species from the same family as oilseed rape (*Brassicaceae*) had higher densities in treated plots and suggested a phylogenetic convergence of weeds [42]. Such specialization of weed assemblages may explain the low effect of herbicides on weed species richness in the centres of oilseed rape fields located in landscapes with low numbers of organic farmed fields and meadows. Conversely, oilseed rape fields in more diversified landscapes may shelter more rare or unspecialized species because of spatial dispersal, explaining the significant effect of herbicides on weed species richness in these fields.

Accounting for landscape in our analysis resulted in an increase of the goodness-of-fit of our models on both weed species richness and abundance in field cores. However, the contribution of landscape variables was low and weed assemblages were generally unaffected by landscape variables. We did however find that a higher numbers of meadows weakened the positive effect of nitrogen on weed species richness. Our results therefore contrast with previous studies conducted in winter cereals, which revealed a positive effect of a higher number of organic farming [28] or seminatural habitats [29] on weed species richness. They are however in accordance with a previous study conducted in oilseed rape fields on the same study site [13], although more generally the literature on the effects of landscape on weed assemblages in oilseed rape fields is still lacking. Although no direct effect of landscape was found, by using structural equations models (SEMs), we revealed a strong indirect effect of landscape on weed assemblages in field cores and field margins. Accounting for the indirect effect greatly increased the goodness-of-fit of the models, especially for weed abundance. We acknowledge that this pattern may arise due to high correlation between weed species richness (or abundance) in field cores and field margins. However, the SEMs showed higher goodness-of-fits with the directionality from field margin towards field core than the contrary. In addition, Bourgeois et al. [13], showed that the similarity of weed assemblages in field core decreased with the distance from the field margin. Therefore, it is likely that landscape affects weed assemblages in the centre of oilseed rape fields across the margins.

Spatial dispersal was the main mechanism shaping weed species richness and abundance in field margins. Among landscape variables, the number of meadows had the strongest effect on weed species richness and abundance. The margins of oilseed rape fields surrounded with a higher number of meadows showed greater weed species richness and weed abundance. The spatial extent of the effects was, however, different: the number of meadows increased weed species richness in field margins at a lower scale (138 m) than weed abundance (266 m). Meadows are seminatural elements of agricultural landscapes that contribute to the maintenance of biodiversity in the agroecosystem by providing food and nesting habitats [43]. Meadows, acting as source habitats, can thus increase weed diversity in field margins. Our results highlighted that spatial dispersal might be the predominant process affecting weeds in field margins, since the variables related to crop competition and farming practices were discarded by the model selection procedure. Such a result is in accordance with our expectations. Indeed, field margins are generally managed at a lower intensity compared to field cores, and crop plants are smaller, present at lower density or even absent. Interestingly, climatic variables had significant effects on weed assemblages in field margins, contrary to field cores. This suggests that in absence of strong filtering factors such as competition or disturbances, climate has a higher filtering effect on weed assemblages. Indeed, a recent study investigating trait–climate relationships in plant assemblages revealed that these relationships were much weaker in croplands compared to grasslands, suggesting a reduced sensitivity of plant assemblages to bioclimatic variations in intensively managed habitats [44].

Our findings suggest that weed assemblages in field cores and margins are shaped by different mechanisms acting at different spatial scales. However, a large part of the variance remained unexplained (around 70% when accounting for dispersal from field margin to field core for both weed richness and abundance). This suggests that other factors may shape weed assemblage such as temporal dispersal [15]. Arable weed species are mainly therophyte species [45], which can persist for long periods as dormant seeds in the seed bank. Such a strategy may allow weed species, and especially those with long, persistent dormant seeds, to avoid unsuitable environmental conditions through delayed emergence (i.e., temporal storage effect, [46]). Further studies should therefore consider the respective roles of temporal dispersal, together with competition, environmental filtering (effect of farming practices) and spatial dispersal.

In conclusion, our study emphasizes the critical importance of crop competition in shaping weed assemblages in field cores, and spatial dispersal in shaping weed assemblages in field margins of oilseed rape fields. Herbicides had a lower effect than crop competition on weed abundance and were shown to reduce weed species richness in oilseed rape fields located in landscapes with higher numbers of extensively managed fields (i.e., organic farmed fields or meadows). Our findings give empirical support for crop competition as a way to reduce the intensity of chemical weeding, and for meadows as a way to enhance biodiversity in agricultural landscapes

## 4. Materials and Methods

### 4.1. Study Area

The study was conducted on the Long-Term Social-Ecological Research (LTSER) site Zone Atelier ‘Plaine & Val de Sèvre’ [47], a 435 km² agricultural landscape located in the Deux-Sèvres district, central western France. Climatic conditions are a mild, temperature, Atlantic oceanic climate (mean annual temperature 12.5 °C and precipitation 867.2 mm). Land use is dominated by cereal production, mostly winter cereals (41.3%), maize (9.6%), sunflower (8.8%) and rapeseed (7.6%). Meadow cover represents around 13%.

### 4.2. Weed Sampling

We surveyed weeds in 115 oilseed rape fields, managed by local farmers, from 2014 to 2018 (23 in 2014, 25 in 2015, 25 in 2016, 20 in 2017 and 22 in 2018). Field size averaged 6.5 ha and ranged from 0.8 ha to 23.1 ha (Electronic supplementary material Table S4). The annual survey spanned from the end of March to late April. Weed flora was monitored in 25 × 1 m^2^ plots per field, each plot being subdivided into four 0.5 × 0.5 m subplots. A total of 20 plots were placed in field core (at least 10 m from the field edge) along two 100 m-long parallel transects (10 plots per transect). The two transects were separated by 50 m and were orthogonal to crop rows. Five plots were placed in the field margin and spaced 10 m apart [48]. We recorded the occurrence of weed species in each subplot and inventoried 157 plant taxa overall (species list in Electronic supplementary material, Table S5).

We computed weed species richness (i.e., the number of species) and abundance separately in the two field compartments. Weed abundance was the sum of individual presence in the 20 or 80 subplots within the field margin and field core, respectively. We did not account for the difference in sampling effort in the study because we conducted the statistical analysis in the two field compartments.

### 4.3. Local, Landscape and Environmental Variables

We used crop height (cm) as a proxy for crop competition because height is related to the plant’s ability to intercept light [49]. During the weed survey, we measured the average canopy height of crop plants in each compartment. In four fields, crop heights in the field margin were missing. We estimated crop height in these fields by averaging the crop height values in fields in which crop height in the field core was 10 cm smaller or greater than the crop height value measured in the field core of the field in which we had a missing observation.

Local management practices related to the level of resources (nitrogen fertilizer) and disturbances (chemical and mechanical weed control) were recorded through farmers’ interviews. The amount of nitrogen input (kg·ha^−1^) was calculated from the fertilizer composition and the quantity applied. The intensity of herbicide applications was assessed using two quantitative indicators [50]: (i) the amount of active substances, which is the sum of the amount of active substances applied, and (ii) the Treatment Frequency Intensity (TFI), which is a measure of the intensity of herbicide application related to the recommended application. The intensity of mechanical weed control was estimated using the average depth of the soil operations. Nitrogen inputs, herbicide applications and mechanical weeding were considered from harvest of the previous crop to the weed sampling date. Data are summarized in the electronic supplementary material (Table S4).

Landscape information was obtained from the land-use database of the LTSER Zone Atelier Plaine & Val de Sèvre [48]. We considered four landscape variables previously shown to affect weed species assemblages, i.e., organic farming [28], seminatural habitats (including meadows and fallows [29,30]), hedgerows measured as a linear [51], but converted of surface of one metre width, and oilseed rape fields [12]. Proportions of landscape variables were computed from the field edge within buffer areas around each field. The scale of buffers for each landscape variable was estimated using the Siland approach [32] which is based on an optimization procedure of the likelihood, without any a priori information on the buffer extent value. For each weed metric, we estimated the buffer radius for the four landscape variables in the two compartments (see below).

### 4.4. Statistical Analysis

The statistical analysis consisted of three main steps. In a first step, we investigated the relative contributions of competition with the crop, resource levels (i.e., amount of nitrogen) and disturbances induced by weed control on weed species richness and abundance in both field cores and field margins. We included crop height and the amount of nitrogen input as proxies of crop competition for resources, and the intensity of herbicide applications and the intensity of soil mechanical operations as proxies for disturbances induced by weed control. To account for interactive effects, we added two-way interactions. Here, we also considered confounding factors acting on weed species richness and abundance, namely field area (in ha), date of sampling (in Julian day as quadratic polynomial), as well as temperature and rainfall, which vary among years. We included temperature (sum of growing degree days, °C) and rainfall (mean precipitation, mm) during the growing period of weeds, rather than throughout the year because these two variables are directly related to plant growth [52,53]. All these variables were included in linear models (LMs) for weed species richness and abundance in the two field compartments (i.e., four LM models were built). We used a variable selection procedure comparing models based on minimizing the Bayesian Information Criterion (BIC [54]) using the dredge function of MuMIn package [55] in R software version 4.0.3 [56]. All explanatory variables involved in at least one of the models with a BIC difference lower than two, from the model with the lowest BIC, were kept for the second step.

In a second step, we examined how the landscape context affects the importance of competition and disturbances on both weed species richness and abundance in field cores and field margins. We built LMs (one for each weed metric in each field compartment) that included the variables retained in the model selection procedure performed in the first step and landscape variables, i.e., hedgerow density and the amount of organic farming, seminatural habitats and oilseed rape fields. We also included the interactions between each landscape variable and the retained variables. The effect and spatial extent of each landscape variable were simultaneously estimated using the Bsiland function of the R package Siland [32]. We used a type III analysis of variance (‘car’ R package, third version [57]).

Finally, the third and last step of the analysis consisted of testing for the effect of local spatial dispersal from the field margin to the field core. We built a Structural Equation Modelling (SEM) where we considered the field margin flora, as an endogenous variable, as well as the variables retained at the second step. Three competing models were tested. The first one incorporated the local and landscape variables included in the linear models built for each metric in step two, without any link relating weed assemblages in the field core and the field margin. In the second model, we tested for an indirect effect of landscape on the weed assemblage in the field core across the field margin. The third model extended the second one by including a direct effect of landscape variables on the weed assemblage in the field core. We considered the strength and directionality of the effect only for the SEM minimizing the BIC, this criterion being relevant to compare SEMs [58]. We assured that the SEMs respected four conditions of well structuration and goodness-of-fit: a *p*-value of the Fischer’s C test > 0.05, a Comparative Fit Index (CFI) > 0.9, a Root Mean Square Error of Approximation (RMSEA) < 0.08 and a Standardized Root Mean Square Residuals (SRMR) < 0.08. We performed SEMs using the package ‘piecewiseSEM’ on R software [59].

All models were run using either one of the two quantitative indicators used to estimate the intensity of herbicide applications, i.e., the amounts of active substances (QA) and the Treatment Frequency Index (TFI). Because the goodness-of-fit of the models using TFI was higher for weed species richness (not for weed abundance) compared to those of the model using QA, only results with TFI are presented here (results with QA are shown in Table S1 and Figures S1–S3). Using TFI and QA generally did not change the general patterns (except for a significant interaction in the weed species richness, see Results section).

When analysing weed abundance in field cores, we found an outlier which affected the outcome of the model. We therefore removed this field from all the analysis conducted with weed abundance (data not shown).

Weed abundance was log10 transformed and explanatory variables were scaled (i.e., transformed using a z-score) using the “scale” function on R software before analysis. We also checked for each model the Variance Inflation Factor (VIF) to control for collinearity between the explanatory variables [60] using the “vif” function in the Car R package [57]. All VIF scores were below 5, showing the absence of problematic collinearity between variables. R-squared values were calculated from the best model determined.

## Figures and Tables

**Table 1:** Statistics of the models for weed (A) species richness and (B) abundance in field cores. Herbicide intensity is expressed with the Treatment Frequency Index (TFI). Abundance was log-transformed, and management practice variables were centred and reduced. The estimated buffer radii are indicated for each landscape variable. Landscape variables have two degrees of freedom because both their spatial extent (i.e., estimation of buffer radius) and their effect were estimated. Significant effects are indicated in bold. R-squared values were 27.4% and 18.0% using TFI.

		Estimated Buffer Radius (m)	Estimate	df	t-Value	*p*-Value
(A) Weed species richness	Intercept		16.314	1	11.204	**<0.001**
	Crop height		−1.407	1	−2.400	**0.018**
	Nitrogen		1.058	1	1.090	0.278
	Herbicides		−1.787	1	−1.788	0.077
	Hedge density	17	48.506	2	1.418	0.159
	Number of meadows	500	−4.439	2	−0.366	0.715
	Amount of organic farming	777	−6.513	2	−0.979	0.330
	Amount of oilseed rape	600	6.913	2	0.714	0.477
	Nitrogen × Herbicides		2.017	1	3.316	**0.001**
	Nitrogen × Number of meadows		−47.839	1	−3.061	**0.003**
	Nitrogen × Amount of organic farming		0.420	1	0.099	0.921
	Herbicides × Number of meadows		−5.072	1	−0.281	0.779
	Herbicides × Amount of organic farming		16.105	1	2.051	**0.043**
(B) Weed Abundance						
	Intercept		2.103	1	42.803	**<0.001**
	Crop Height		−0.088	1	−3.101	**0.002**
	Herbicides		−0.075	1	−2.223	**0.028**
	Hedge density	68	6.262	2	1.345	0.181
	Number of meadows	26	−0.167	2	−0.429	0.669
	Amount of organic farming	140	0.159	2	0.814	0.418
	Amount of oilseed rape	26	0.199	2	1.075	0.285
	Herbicides × Number of meadows		0.938	1	1.674	0.097
	Herbicides × Amount of organic farming		0.353	1	1.394	0.166

**Table 2:** Statistics of the models for weed (A) species richness and (B) abundance in field margins. Abundance was log-transformed, and environmental variables were centred and reduced. The estimated buffer radii are indicated for each landscape variable. Landscape variables have two degrees of freedom because both their spatial extent and their effect were estimated. Significant effects are indicated in bold. R-squared of respective models computed with type III ANOVAs were 12.3% and 22.7%.

(A)		Estimated Buffer Radius (m)	Estimate	df	t-Value	*p*-Value
	Intercept		20.262	1	10.818	**<0.001**
	Rainfall		−1.805	1	−2.467	**0.015**
	Hedge density	37	−86.517	2	−1.083	0.281
	Number of meadows	138	18.731	2	2.184	**0.031**
	Amount of organic farming	20	8.255	2	1.642	0.103
	Amount of oilseed rape	980	−11.572	2	−0.711	0.478
**(B)**						
	Intercept		1.892	1	60.673	**<0.001**
	Rainfall		−0.087	1	−4.060	**<0.001**
	Temperature		0.051	1	2.310	**0.023**
	Hedge density	5	−0.990	2	−1.892	0.061
	Number of meadows	266	0.664	2	1.991	**0.049**
	Amount of organic farming	22	0.168	2	1.244	0.216
	Amount of oilseed rape	8	−0.099	2	−0.755	0.452

## Data Availability

Data will be provided upon request to the corresponding author.

## Supplementary Materials

The following are available online at https://www.mdpi.com/article/10.3390/plants10102131/s1: Figure S1: Percentage of variance explained by local, landscape and environmental variables when using the quantity of active substances as a proxy for herbicide intensity; Figure S2: Model predictions for weed abundance in field cores using the quantity of active substances as a proxy for herbicide intensity; Figure S3: Structural Equation Models for weed species richness (A) and abundance (B) using the quantity of active substances; Table S1: Statistics of the models of weed (A) species richness and (B) abundance in field core using the quantity of active substances; Table S2: Statistics of the competing structural equation models with the type of link specified between field margin and field core floras using the quantity of active substances; Table S3: Statistics of the competing structural equation models with the type of link specified between field margin and field core floras using the treatment frequency intensity; Table S4: Descriptive statistics for local and environmental variables, Table S5: List of the taxa identified in the 115 oilseed rape fields of the study.
