# Supplementary material for: Landscape Is the Main Driver of Weed Assemblages in Field Margins but Is Outperformed by Crop Competition in Field Cores
Source: Plants (Basel). 2021 Oct 7;10(10):2131. doi: 10.3390/plants10102131 (PMC8537063; doi:10.3390/plants10102131)
Supplement: Supplementary file 1 [file plants-10-02131-s001.zip › plants-1362317-supplementary.pdf]

Table S1. Statistics of the models for weed (A) species richness and (B) abundance in field cores. Herbicide intensity is expressed with the quantity of active substance (QA). Abundance was log-transformed, and management practice variables were centered and reduced. The estimated buffer radii are indicated for each landscape variable. Landscape variables have two degrees of freedom because both their spatial extent (i.e. estimation of buffer radius) and their effect were estimated. Significant effects are indicated in bold. R-squared were 22.4% and 16.0% for weed species richness and abundance.

		**Estimated buffer radius (m)**	**Estimate**	**df**	**t-value**	**p-value**
(A) Weed species richness						
	Intercept		17.394	1	14.018	**<0.001**
	Crop height		-1.403	1	-2.389	**0.019**
	Nitrogen		0.886	1	0.928	0.356
	Herbicides		-1.193	1	-1.225	0.223
	Hedge density	17	38.128	2	1.114	0.268
	Amount of meadows	586	-2.332	2	-0.212	0.833
	Amount of organic farming	739	-10.045	2	-1.423	0.158
	Amount of oilseed rape	209	-4.164	2	-0.806	0.422
	Nitrogen × Herbicides		1.328	1	2.433	**0.017**
	Nitrogen × Amount of meadows		-39.328	1	-2.622	**0.010**
	Nitrogen × Amount of organic farming		-0.432	1	-0.056	0.956
	Herbicides × Amount of meadows		-15.245	1	-0.922	0.359
	Herbicides × Amount of organic		12.966	1	1.946	0.054
(B) Weed abundance						
	Intercept		2.103	1	43.069	**<0.001**
	Crop Height		-0.084	1	-2.953	**0.004**
	Herbicides		-0.066	1	-2.175	**0.032**
	Hedge density	68	6.351	2	1.361	0.177
	Amount of meadows	1	-0.590	2	-1.263	0.209
	Amount of organic farming	28	0.299	2	1.449	0.150
	Amount of oilseed rape	25	0.164	2	0.906	0.367
	Herbicides × Amount of meadows		0.239	1	0.373	0.710
	Herbicides × Amount of organic		-0.011	1	-0.062	0.950

Table S2. Statistics of the competing structural equation models with different types of link between field margin and field core floras. 'Indirect link' tests the effect of landscape variables on field core flora through the endogenous variable (either weed species richness or abundance in field margin), while 'direct link' tests the effects of landscape variables directly on the field core flore. 'No link' indicates that floras in field core and field margin were not related. Herbicide intensity is expressed with the Quantity of Active substances (QA). Endogenous variables of each model are written in italic. R-squared for each response or endogenous variable, and the BIC of the structural equation model are presented. FM: Field margin and FC: Field core.

Competing mechanism	Response variables	Model description	R^2	BIC
Indirect link	Richness FC	*RichnessFM* + Nitrogen + Crop Height + Herbicides (QA) + Nitrogen x Herbicides	33	75.4
	Richness FM	Meadows + Organic + Hedges + Oilseed rape + Rainfall	12	
Direct link	Richness FC	*RichnessFM* + Nitrogen + Crop Height + Herbicides (QA) + Nitrogen x Herbicides (QA) + OrganicFM + MeadowsFM + HedgesFM + Oilseed rapeFM	34	90.4
	Richness FM	Meadows + Organic + Hedges + Oilseed rape + Rainfall	12	
No link	Richness FC	Nitrogen + Crop Height + Herbicides (QA) + Nitrogen x Herbicides (QA)	17	97.9
	Richness FM	Meadows + Organic + Hedges + Oilseed rape + Rainfall	12	
Indirect link	Abundance FC	*Abundance FM* + Crop Height + Herbicides (QA)	32	67.3
	Abundance FM	Meadows + Temperature + Organic + Hedges + Oilseed rape + Rainfall	21	
Direct link	Abundance FC	*Abundance FM* + Crop Height + Herbicides (QA) + OrganicFM + MeadowsFM + HedgesFM + Oilseed rapeFM	33	82.5
	Abundance FM	Meadows + Temperature + Organic + Hedges + Oilseed rape + Rainfall	21	
No link	Abundance FC	Crop Height + Herbicides (QA)	11	97.0
	Abundance FM	Meadows + Temperature + Organic + Hedges + Oilseed rape + Rainfall	21	

Table S3. Statistics of the competing structural equation models with different types of link between field margin and field core floras. 'Indirect link' tests the effect of landscape variables on field core flora through the endogenous variable (either weed species richness or abundance in field margin), while 'direct link' tests the effects of landscape variables directly on the field core flore. 'No link' indicates that floras in field core and field margin were not related. Herbicide intensity is expressed with the Treatment Frequency Index (TFI). Endogenous variables of each model are written in italic. R-squared for each response or endogenous variable, and the BIC of the structural equation model are presented. FM: Field margin and FC: Field core.

Competing mechanism	Response variables	Model description	R^2	BIC
Indirect link	Richness FC	*RichnessFM* + Nitrogen + Crop Height + Herbicides (TFI) + Nitrogen x Herbicides	30	76.5
	Richness FM	Meadows + Organic + Hedges + Oilseed rape + Rainfall	12	
Direct link	Richness FC	*RichnessFM* + Nitrogen + Crop Height + Herbicides (TFI) + Nitrogen x Herbicides + OrganicFM + MeadowsFM + HedgesFM + Oilseed rapeFM	32	90.9
	Richness FM	Meadows + Organic + Hedges + Oilseed rape + Rainfall	12	
No link	Richness FC	Nitrogen + Crop Height + Herbicides (TFI) + Nitrogen x Herbicides	15	95.9
	Richness FM	Meadows + Organic + Hedges + Oilseed rape + Rainfall	12	
Indirect link	Abundance FC	*Abundance FM* + Crop Height + Herbicides (TFI)	30	68.1
	Abundance FM	Meadows + Temperature + Organic + Hedges + Oilseed rape + Rainfall	21	
Direct link	Abundance FC	*Abundance FM* + Crop Height + Herbicides (TFI) + OrganicFM + MeadowsFM + HedgesFM + Oilseed rapeFM	31	82.3
	Abundance FM	Meadows + Temperature + Organic + Hedges + Oilseed rape + Rainfall	21	
No link	Abundance FC	Crop Height + Herbicides (TFI)	9	96.4
	Abundance FM	Meadows + Temperature + Organic + Hedges + Oilseed rape + Rainfall	21	

Table S4. a) Descriptive statistics for local and environmental variables. b) List of the herbicide molecules applied the studied fields.

a)

	Mean (±SD)	Min.	Max.
Crop height in field core (cm)	145.9 (±23.7)	80.0	190.0
Crop height in field margin (cm)	55.8 (±47.8)	0.0	170.0
Nitrogen input ($kg.ha^{-1}$)	167.7 (±55.4)	35.1	323.9
Herbicide active quantity ($g.\ ha^{-1}$)	1670.0 (±910.0)	0.0	4123.0
Treatment Frequency Index	1.9 (±1.1)	0.0	5.8
Mean tillage depth (cm)	7.4 (±2.7)	0.0	17.5
Sampling date (Julian day)	104.7 (±6.3)	84.0	114.0
Sum of mean growing day temperature (°C)	508.4 (±128.9)	302.0	689.3
Mean of growing day rainfall quantity (mm)	2285.0 (±239.0)	1702.0	2783.0
Field area (ha)	6.6 (±4. 7)	0.8	23.1

b) List of the herbicide molecules applied in the studied fields

α-chloroacetamides
acetamides
aryloxyphenoxy-propionates (fops)
benzamides
cyclohexanediones (dims)
diphenyl ether
glycine
imidazolinones
isoxazolidinone
phenoxy-carboxylates
phenyl ethers
phenylpyrazoline
pyridine-carboxylates
quinoline-carboxylates
sulfonylureas
triazolopyrimidine - type 1
triketones

Table S5. List of the taxa identified in the 115 oilseed rape fields of the study, according to the nomenclature used in Flora Gallica (Tison, Jean Marc & de Foucault, Bruno, 2014. Flora Gallica. Flore de France. Biotope, Mèze, 1196p). "Family sp" is used to refer to plants that could not be identified at the species level. We indicate the frequency of occurrence of the species in the whole data (%), and in fields (%).

Species name	Frequency of occurrence (%)		Species name	Occurrence (%)	
	Observations	Fields		Observations	Fields
Achillea millefolium L.	0.01	1.74	*Carduus* (L.) *sp*	0.20	18.26
Adonis annua L.	0.05	2.61	*Centaurea jacea* L.	0.02	2.61
Aethusa cynapium L.	0.09	7.83	*Cerastium glomeratum* Thuill.	0.18	13.91
Alliaria petiolata (M. Bieb.) Cavara & Grande	0.06	3.48	*Chenopodiastrum hybridum* (L.) S.Fuentes, Uotila & Borsch	0.02	1.74
Allium oleraceum L.	0.16	8.70	*Chenopodium album* L.	0.89	33.91
Alopecurus myosuroides Huds. *subsp. myosuroides*	4.24	66.96	*Cirsium arvense* (L.) Scop.	1.21	56.52
Althaea hirsute L.	0.01	0.87	*Cirsium vulgare* (Savi) Ten.	0.18	9.57
Ammi majus L.	0.67	20.00	*Clematis vitalba* L.	0.02	0.87
Anisantha sterilis (L.) Nevski	0.81	39.13	*Convolvulus arvensis* L.	4.30	83.48
Anthemis arvensis L.	0.00	0.87	*Crepis sancta* (L.) Bornm.	0.20	18.26
Anthemis cotula L.	0.27	1.74	*Cyanus segetum* Hill	0.05	2.61
Anthriscus caucalis M.Bieb.	0.08	6.96	*Dactylis glomerata* L.	0.02	3.48
Anthriscus sylvestris (L.) Hoffm.	0.02	1.74	*Daucus carota* L.	0.87	45.22
Aphanes arvensis L.	1.45	31.30	*Draba muralis* L.	0.04	3.48
*Apiaceae (*Lindl.) *sp.*	0.00	0.87	*Echinochloa crus-galli* (L.) P.Beauv.	0.02	1.74
*Arabidopsis thaliana (*L.) Heynh.	0.01	0.87	*Elytrigia repens* (L.) Desv. ex Nevski	0.47	21.74
Arctium lappa L.	0.05	3.48	*Epilobium tetragonum* L.	0.10	8.70
Arenaria serpyllifolia L.	0.12	9.57	*Erigeron* L. *sp*	0.00	0.87
Arrhenatherum elatius (L.) J. Presl & C. Presl	0.02	3.48	*Erodium cicutarium* (L.) L,Hér.	0.62	10.43
Arum italicum Mill.	0.04	5.22	*Erodium moschatum* (L.) L,Hér.	0.01	1.74
*Asteraceae (*Martinov) *sp.*	0.02	2.61	*Euphorbia helioscopia* L.	1.13	43.48
Atriplex patula L.	1.25	41.74	*Euphorbia peplus* L.	0.01	1.74
Avena fatua L.	1.73	49.57	*Falcaria vulgaris* Bernh.	0.24	23.48
Barbarea intermedia Boreau	0.01	1.74	*Fallopia convolvulus* (L.) Á.Löwe	5.98	78.26
Borago officinalis L.	0.01	0.87	*Festuca rubra* L.	0.17	8.70
Brassicaceae (Burnett.) *sp*	0.01	1.74	*Ficaria verna* Huds.	0.03	3.48
Bromus hordeaceus L.	0.72	32.17	*Fumaria officinalis* L.	0.19	16.52
Bryonia dioica Jacq.	0.01	0.87	*Galium aparine* L.	4.37	84.35
Buglossoides arvensis (L.) I.M.Johnst.	0.01	1.74	*Galium mollugo* L.	0.07	10.43
Calepina irregularis (Asso) Thell.	0.60	18.26	*Geranium columbinum* L.	0.16	13.91
Capsella bursa pastoris (L.) Medik.	0.94	37.39	*Geranium dissectum* L.	4.02	79.13
Cardamine hirsute L.	0.03	3.48	*Geranium molle* L.	1.67	51.30

Geranium pusillum L.	0.05	4.35	*Plantago lanceolata* L.	0.10	13.04
Geranium robertianum L.	0.31	15.65	*Poa annua* L.	0.46	15.65
Geranium rotundifolium L.	0.62	24.35	*Poa pratensis* L.	0.03	3.48
Glechoma hederacea L.	0.00	0.87	*Poa trivialis* L.	0.35	20.87
Hedera helix L.	0.01	1.74	*Polygonum aviculare* L.	4.23	68.70
Helianthus annuus L.	0.00	0.87	*Potentilla reptans* L.	0.07	6.96
Heliotropium europaeum L.	0.01	1.74	*Prunella vulgaris* L.	0.01	1.74
Helminthotheca echioides (L.) Holub	0.82	53.91	*Ranunculus parviflorus* L.	0.07	6.96
Hypericum perforatum L.	0.03	1.74	*Raphanus raphanistrum* L.	0.16	3.48
Hypochaeris radicata L.	0.00	0.87	*Reseda lutea* L.	1.33	38.26
Jacobaea vulgaris (Gaertn.) *subsp. vulgaris*	0.01	1.74	*Rubus fruticosus* L.	0.27	24.35
Kickxia spuria (L.) Dumort.	0.44	15.65	*Rumex crispus* L.	0.16	13.04
Knautia arvensis (L.) Coult.	0.00	0.87	*Rumex obtusifolius* L.	0.02	0.87
Lactuca serriola L.	0.15	24.35	*Saxifraga tridactylites* L.	0.00	0.87
Lactuca virosa L.	0.02	1.74	*Scandix pecten veneris* L.	0.22	11.30
Lamium amplexicaule L.	0.23	10.43	*Schedonorus arundinaceus (*Schreb.) Dumort.	0.08	2.61
Lamium purpureum L.	0.94	49.57	*Schedonorus pratensis (*Huds.) P.Beauv.	0.04	1.74
Lapsana communis L.	0.23	15.65	*Senecio vulgaris* L.	0.66	39.13
Lathyrus aphaca L.	0.03	3.48	*Sherardia arvensis* L.	0.68	40.00
Legousia speculum-veneris (L.) Chaix	0.20	7.83	*Silene latifolia* Poir. *subsp. alba*	0.96	45.22
Linaria vulgaris Mill.	0.01	1.74	*Silene vulgaris* (Moench) Garcke	0.03	4.35
Lolium multiflorum Lam.	1.89	40.87	*Silybum marianum (*L.) *Gaertn.*	0.23	15.65
Lysimachia arvensis (L.) U.Manns & Anderb.	3.89	44.35	*Sinapis alba* L.	0.27	6.09
Malva sylvestris L.	0.65	27.83	*Sinapis arvensis* L.	2.19	50.43
Matricaria chamomilla (L.) Bernh.	0.06	4.35	*Sison segetum* L.	0.15	13.04
Medicago arabica (L.) Huds.	0.04	1.74	*Sisymbrium officinale (*L.) Scop.	0.36	6.96
Medicago lupulina L.	0.04	6.09	*Solanum nigrum* L.	0.46	17.39
Medicago minima (L.) L.	0.00	0.87	*Sonchus asper* (L.) Hill	2.29	55.65
Medicago polymorpha L.	0.04	2.61	*Sonchus oleraceus* L.	1.10	50.43
Medicago sativa L.	0.24	4.35	*Stachys arvensis* (L.) L.	0.03	5.22
Mentha suaveolens Ehrh.	0.01	0.87	*Stellaria media* (L.) Vill.	1.30	26.09
Mercurialis annua L.	7.40	80.00	*Symphytum officinale* L.	0.00	0.87
Muscari comosum (L.) Mill.	0.05	6.96	*Taraxacum* F.H. Wigg. *sp.*	0.14	14.78
Myosotis arvensis (L.) Hill	0.81	34.78	*Thlaspi arvense* L.	0.05	5.22
Ononis spinosa L.	0.02	1.74	*Tordylium maximum* L.	0.03	4.35
Ornithogalum umbellatum L.	0.01	0.87	*Torilis arvensis* (Huds.) Link	0.84	37.39
Papaver rhoeas L.	1.68	65.22	*Torilis nodosa* (L.) Gaertn.	0.00	0.87
Papaver somniferum L. *subsp. somniferum*	0.04	0.87	*Trifolium incarnatum* L.	0.00	0.87
Persicaria maculosa Gray	0.28	5.22	*Trifolium pratense* L.	0.01	1.74
Phacelia tanacetifolia Benth.	0.02	1.74	*Trifolium repens* L.	0.02	2.61
Picris hieracioides L.	1.32	54.78	*Tripleurospermum inodorum* (L.) Sch. Bip.	0.65	7.83

Triticum L. *sp*	2.92	65.22	*Vicia faba* L.	0.10	1.74
Valerianella locusta (L.) Laterr.	0.24	24.35	*Vicia sativa* L.	0.26	8.70
Veronica arvensis L.	0.61	26.96	*Vinca minor* L.	0.02	1.74
Veronica hederifolia L.	2.74	63.48	*Viola arvensis* Murray	5.79	79.13
Veronica persica Poir.	3.35	73.91	*Vulpia bromoides* (L.) Gray	0.06	2.61
Veronica polita Fr.	0.05	2.61	*Vulpia myuros* (L.) C.C.Gmel	0.01	0.87

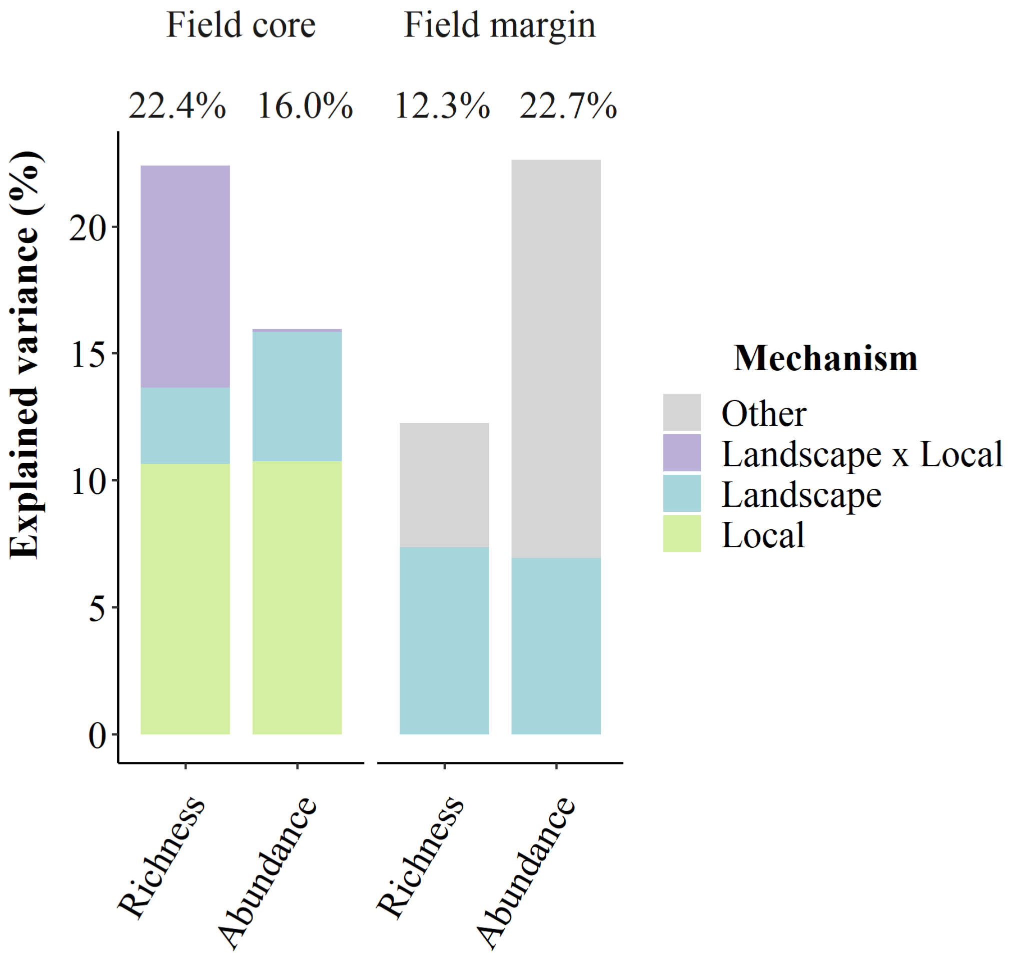

Figure S1: Percentage of explained variance by local factors (crop height, the amount of nitrogen, the intensity of herbicides use and number of mechanical operations), landscape (amount of organic farming, meadows, oilseed rape and hedge density) and weather conditions (rainfall and temperature) on weed species richness and abundance in field cores and field margins. The intensity of herbicide use is expressed using the quantity of active substances. The buffer radii at which the amount of each landscape variable was estimated are shown in Table 1 for field cores and Table 2 3 for field margins. R-squared computed from the type III Anovas of respective models are indicated above each corresponding barplot.

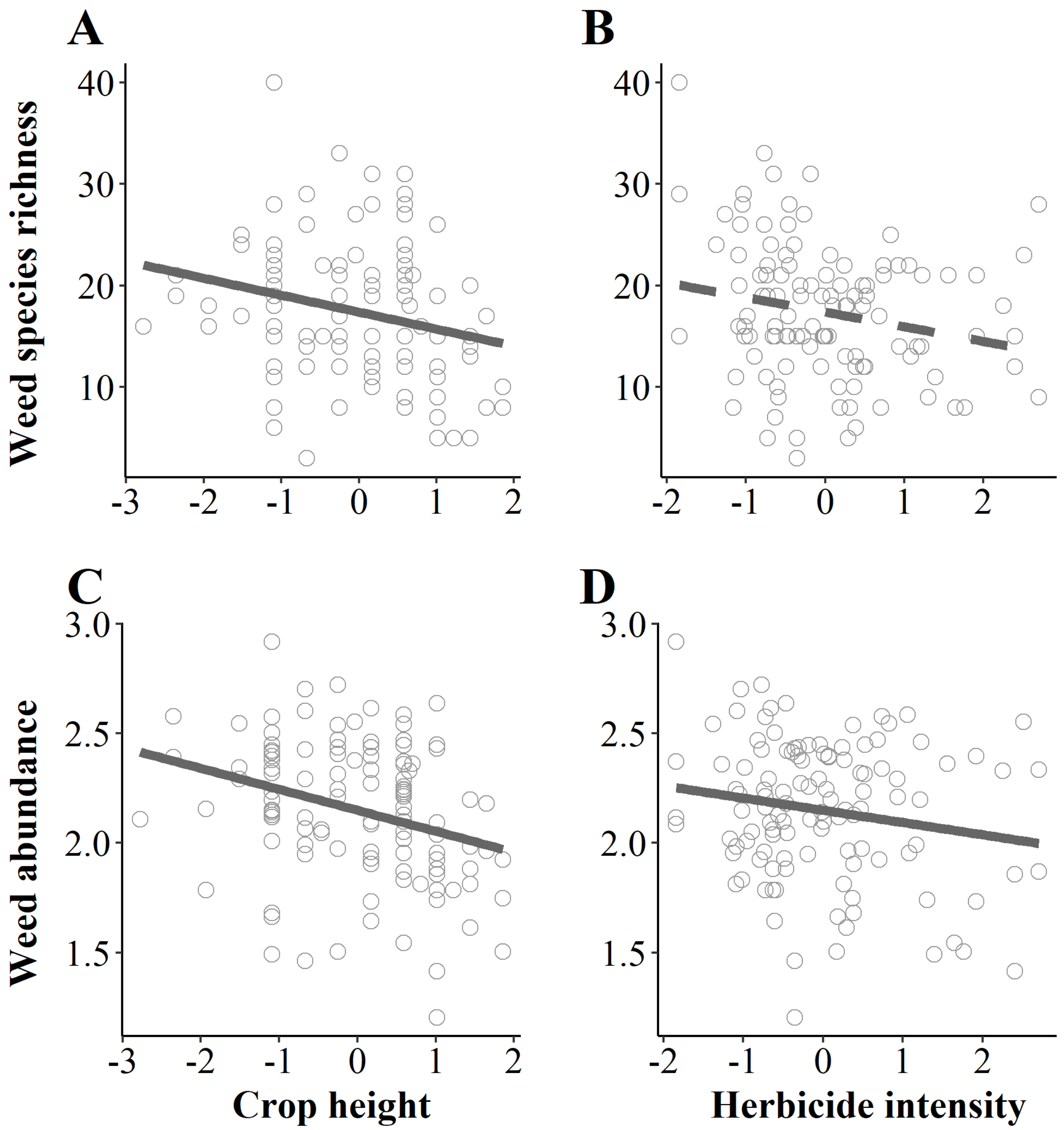

Figure S2: Relationship between weed species richness (A,B) and abundance (C,D) in field cores with crop height (A,C) and the intensity of herbicide applications (B,D). Abundance was log-transformed and explanatory variables were scaled. The intensity of herbicide use is expressed using the Quantity of Active substances (QA). Dashed lines indicate non-significant relationships.

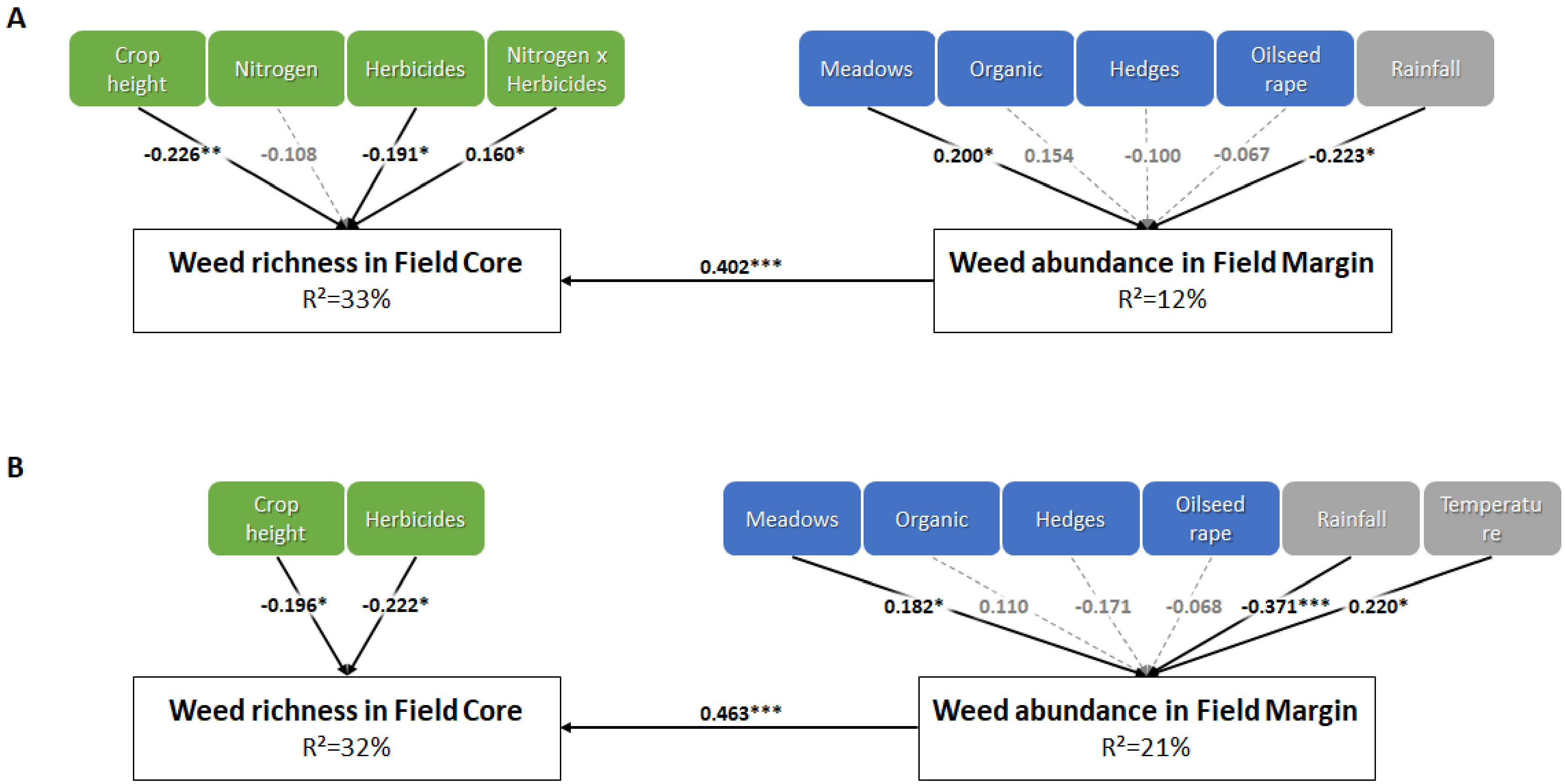

Figure S3. Structural Equation Models for weed species richness (A) and abundance (B) where the link be-tween field margin and field core was specified. Arrows represent the directionality of the effect, and the coefficients indicate the standardized estimates. Dashed lines represent non-significant effects. *: p-value < 0.05; **: p-value < 0.01; ***: p-value <0.001The intensity of herbicide use is expressed using the Quantity of Active substances. The buffer radius at which the amount of each landscape variable was estimated is shown in Table 2 for field margin.
